# Prevalence of Sarcopenia in Community-Dwelling Older Adults in Valencia, Spain

**DOI:** 10.3390/ijerph17239130

**Published:** 2020-12-07

**Authors:** Carlos Guillamón-Escudero, Angela Diago-Galmés, Jose M. Tenías-Burillo, Jose M. Soriano, Julio J. Fernández-Garrido

**Affiliations:** 1 Hospital General Universitario de Castelló, 12004 Castellón, Spain; carlos_ge@hotmail.es; 2 Hospital Universitario de La Plana, 12540 Villareal, Spain; angela94dg@gmail.com; 3 Department of Preventive Medicine, Hospital Pare Jofré, 46017 Valencia, Spain; Tenias_jma@gva.es; 4 Food & Health Lab, Institute of Materials Science, University of Valencia, 46980 Valencia, Spain; 5 Joint Research Unit on Endocrinology, Nutrition and Clinical Dietetics, University of Valencia-Health Research Institute La Fe, 46026 Valencia, Spain; 6 Department of Nursing, Faculty of Nursing and Podiatry, University of Valencia, 46001 Valencia, Spain; Julio.Fernandez@uv.es

**Keywords:** sarcopenia, prevalence, older adults, European Working Group on Sarcopenia in Older People 2 (EWGSOP2), older people, muscle strength

## Abstract

This study is an observational and cross-sectional study on the prevalence of sarcopenic disease in 202 autonomous older adults; 18.8 and 81.2% were men and women, respectively, living in their own homes in Valencia, Spain. Sarcopenia was diagnosed using the criteria and cutting points for the European Working Group on Sarcopenia in Older People 2 (EWGSOP2), using the tests: SARC-F, grip strength, sit-to-stand, gait speed, appendicular skeletal muscle mass and short physical performance battery. According to the EWGSOP2 criteria, probable sarcopenia was present in 21.1% and 18.3% of men and women, respectively, and the sum of confirmed and severe sarcopenia was 7.9% and 7.3% in men and in women, respectively. A relationship was shown between the prevalence of the disease and the age of the participants, but no significant differences were found between the sum of confirmed and severe sarcopenia between the sexes, nor a relationship between the amount of muscle mass and the strength of grip. The SARC-F questionnaire diagnosed 40% of the sarcopenia cases present in the study. More thorough research is needed to continue using the EWGSOP2 criteria in different populations to establish a correct prevalence of sarcopenic disease in different populations of the world.

## 1. Introduction

Sarcopenia has originally been defined as the loss of muscle mass due to aging [1,2,3]. However, during the last decade, the term has acquired complexity and has been used to define the loss of strength and muscle mass in relation to aging [4,5]. In fact. changes in muscle mass associated with force implies accepting the existence of a direct relationship between both factors, affirming that changes in muscle mass are responsible for changes in strength [6,7,8], suggesting that aging over 65 years of age, combined with a deterioration of muscle strength, have a negative influence in the construction of muscle mass and its quality.

Furthermore, muscle mass deficit alone is not a good predictor of impaired functionality and mortality due to the fact that the loss of strength has been associated with these results in multiple studies [9,10,11,12], and based on these results, the concept of dynapenia appears, which is defined as the loss of age-related force whose etiology is not of neurological or muscular origin [7,8]. According to the consensus of the European Working Group on Sarcopenia in Older Persons (EWGSOP) establishes that the diagnosis of sarcopenia is based on the loss of muscle mass necessarily associated with strength and physical performance [3] being demonstrated as a good predictor of disability and early mortality [13,14]. In 2019, the EWGSOP [3], following its previous consensus published in 2010 [15], presented the new revised guidelines for the definition and diagnosis of sarcopenia with a significant variation in the algorithm for diagnosis of the disease, increasing the relevance of muscle strength in the diagnosis of sarcopenia and modifying the cutoff points that determined the diagnosis of the disease in most of the complementary tests used. In this context, it becomes essential to update the evidence related to the prevalence of sarcopenia in the world, since after updating the diagnostic criteria of the EWGSOP 2019 [3], all the accumulated previous evidence on this subject is outdated and no longer corresponds to the real analysis of the disease and how it affects the population. In Spain, these studies have been still underdeveloped and it will surely be a major problem in the years to come given the diagnostic difficulty of sarcopenia, the volatility in the information published about it, the aging of the population, and the increase in physical inactivity [16]. The aim of this study was to demonstrate the prevalence of sarcopenic disease in autonomous older adults who are neither hospitalized nor residing in nursing homes in Valencia, Spain.

## 2. Materials and Methods

### 2.1. Study Population

A cross-sectional descriptive study was carried out in over-65s attending municipal activity centers for the elderly, which is integrated into the framework of the Chair of Healthy, Active and Participative Aging signed between the University of Valencia and the City Council of Valencia. The participation was voluntary. None of the older participants were hospitalized during the study and all resided in their private homes. Participants were informed of this study, which was in accordance with the fundamental principles of the Declaration of Helsinki. This study was approved by the Ethical Committee of University of Valencia (Spain) [17]. In order to avoid considering cases of sarcopenia secondary to other pathologies and whose inclusion in the study could have interfered with the analysis of the study, all those whose score in the Barthel test [18] would have been less than 60 points were excluded, which ensured that all were independent or slightly dependent on basic activities of daily living (ADLs). 

The following inclusion criteria were applied for all participants: (a) be over 65 years of age; (b) be registered at the Senior Citizens Centre; (c) have a Barthel Index equal to or greater than 60 points; (d) complete the consent and ability to understand and complete all tests included in the study. 

In order to carry out this publication, after consulting the recent work carried out by different authors [19,20,21] on the prevalence of sarcopenia in noninstitutional older patients, it was determined that this variable ranged from 10% to 15%. Therefore, admitting a degree of error in the estimate of 4% to 5%, and with a confidence interval of 95%, it was calculated as necessary to recruit from 139 (case of lower prevalence and precision) to 307 subjects (case of higher prevalence and precision) to obtain a stable and statistically reliable sample. Thus, a sample size that exceeds two hundred cases, like that of this study, allows us to make an acceptable estimate of the prevalence of sarcopenia. The sample size calculations were done with the EPIDAT 4.2 software.

### 2.2. Examination Protocol and Measurements

The general information of the participants was collected through an ad hoc questionnaire, administered by the researchers. The variables collected were age and sex, the type of cohabitation in the home and the illnesses suffered by the participants. 

### 2.3. Degree of Dependence

The Barthel index was used to determine the degree of dependence of the study participants. Validated scale for Spanish population [18] and of wide clinical use [22,23,24], which considers as independent the subjects who, after the performance of the index, obtain a score of 100. This study included subjects who scored between 60 (mild dependence degree) and 100 (total independent), since none of these groups presented significant difficulties in the realization of (ADLs).

### 2.4. Sarcopenia Screening

In addition to the study and solely for the purpose of testing the reliability of the SARC-F questionnaire, it was completed by all subjects regardless of whether or not sarcopenia was suspected. The SARC-F questionnaire [25] has been proposed by EWGSOP 2019 [3] for universal sarcopenia screening. This test consists of 5 sections in which the perceived force of the participant is evaluated, the use of devices to walk, the difficulty to get up from a chair and to climb stairs, and the frequency with which they suffer falls. A score ≤4 was considered susceptible to sarcopenia, although it had no practical effect on the subsequent diagnostic decision.

### 2.5. Diagnosis of Sarcopenic Pathology

Recommendations of the EWGSOP [3] were used for the determination of the sarcopenic pathology of the subjects studied, which integrates three dimensions: (i) low muscle strength [11], (ii) low muscle quantity or quality [26] and (iii) poor physical performance [27]. These dimensions were analysed individually with the corresponding previously validated tool. In addition, the SARC-F questionnaire was used to assess the ability to quickly identify cases of sarcopenia [25]. Following the criteria of the EWGSOP 2019 [3], those individuals who had only low muscle strength, both in the lower and upper trains, were considered as probably sarcopenia [3]; if along with this deterioration of muscle strength there was also evidence of a low quantity or quality of muscle mass, the participant was classified as “confirmed sarcopenia”; and, lastly, if in addition to these two variables, the subjects had a decrease in physical performance, sarcopenia was classified as probable, confirmed or severe sarcopenia.

### 2.6. Grip Force (Upper Train)

To determine the force of the upper train, manual ergometry [12,28,29,30] measured with the analogue hydraulic hand dynamometer Jamar 5030J1 was used, with a measuring scale of 0–90 kg/force (kg/f) and an accuracy of 2 kg. The protocol for taking measurements consisted of two attempts, with each hand, to perform the maximum of voluntary grip force. Between each of them, a one-minute break was left, and the highest score obtained among the four total attempts was taken as the final value. The measurements were made with the participants seated in a chair with a straight back, the arm bent at an angle of 90 degrees and being in contact with the trunk. During the measurement, the arm under study was not supported on any surface [31,32]. Values of <27 and <16 kg in men and women, respectively, were considered as indicative of a decrease in force.

### 2.7. Lower Train Strength

To determine the force in the lower train, the test called “sit-to-stand” of 5 repetitions was used, for its practicality and simplicity. This test is included in the test battery proposed by the EWGSOP [3] and consisted of making 5 squats to the chair, at the maximum possible speed and without using any type of manual support [33]. To evaluate the test result, the time used by the subjects in the development of the test was taken into account. Values greater than 15 s were considered as indicative of a decrease in the strength of the participants, regardless of sex.

### 2.8. Appendicular Skeletal Muscle Mass (ASMM)

For the determination of ASMM, indispensable for the categorization of sarcopenia cases, the equation proposed by Kyle et al. [34] was used. In order to develop this formula, we used the results obtained through the electrical bioimpedance carried out with a calibrated digital scale (TANITA DC 430MA-S, Tokyo, Japan; with an accuracy of 0.05 kg) following the latest existing recommendations [35]. Values of <20 and 15 kg in men and women, respectively, were considered indicative of muscle mass decrease (deficit), according to the classification established by the EWGSOP [3].

### 2.9. Physical Performance

The physical performance of the participants was measured by the 4 m speed test [15,36] and, in addition to the “Short Physical Performance Battery” test (SPPB test), following recommendations proposed by the EWGSOP [3]. The first test consisted of measuring the time it took participants to walk a distance of 4 m, at the usual speed, and values of less than 0.8 m/s, regardless of sex, were considered as indications of impairment of physical performance. The SPPB test consists of three tests to evaluate the balance (participants carried out three positions, i.e., standing with their feet as close together, after semi-tandem and finally tandem position, for 10 s in each position), speed (studied population developed to travel 4 m at a usual pace) and force (being pretested to fold their arms across their chest and stand up from the chair; if the pretest was carried out, participants must perform five chair stands as quickly as possible) [37,38]. The scoring and evaluation of the total result results from the sum of the scores obtained in the three tests. Values of ≤8 points, regardless of sex, were considered as indications of impairment of physical performance.

### 2.10. Statistical Analysis

Statistical analysis was done with IBM SPSS Statistics v.24 (IBM Corp., Armonk, NY, USA) software for Windows. The Kolmogorov–Smirnov normality test was used to check the normal distribution of data and variables. Student *t* statistic and the U–Mann statistic–Whitney were carried out when normality was found in the variables and when we determined an abnormal distribution, respectively. Quantitative variables were presented as means and standard deviations, being analysed with the Fisher’s test statistic; while qualitative variables were presented as relative frequencies and percentages.

## 3. Results

**Figure 1:**
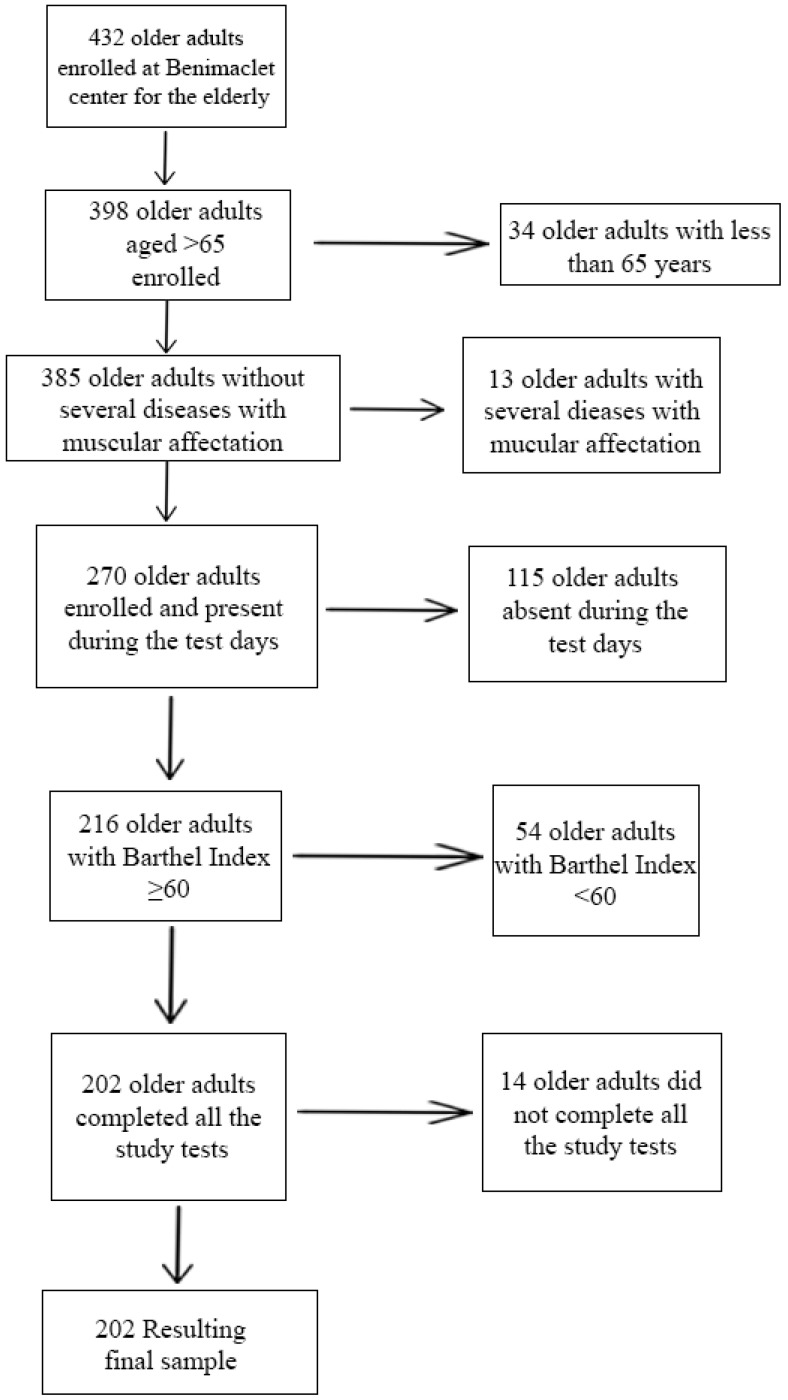
Flow diagram for recruitment of older adults for participation in this study.

**Figure 2:**
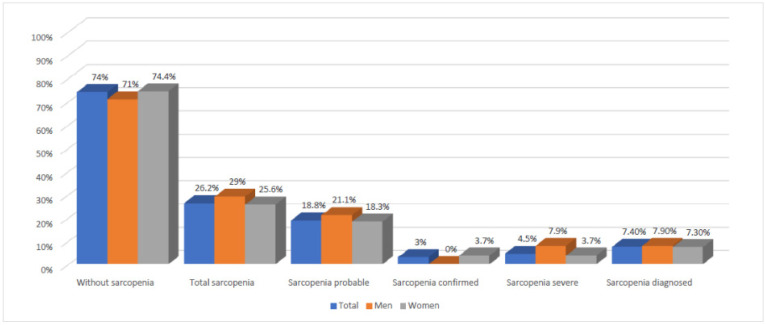
Categorized prevalence of sarcopenia in community-dwelling older adults according to EWGSOP 2019 diagnostic criteria and gender.

The initial sample included 432 elderly persons of both sexes, of whom 34 were excluded as being under 65 years of age. Subjects who had a disease involving severe impairment of muscle mass (n = 13), those who did not have a Barthel equal to or greater than 60 (n = 54), those who were not present on the days of study (n = 115) and those who did not complete the study (n = 14) were not included in the final sample. The participation rate was 46.76% and the final sample was 202 people (Figure 1), 18.8% (38 subjects) and 81.2% (164) being men and women, respectively. In fact, influx in the studied place is higher than men. The average age of the participants in the study was 73.01 years; the youngest was a woman of 65 years and the oldest was an 85-year-old woman. Of the males, 71.5% were between 65 and 75 years of age and 28.95% between 75 and 85 years of age. Women aged 65 to 75 years and 75 to 85 years old were 61.59 and 38.41%, respectively. Of the total sample, 73.8% (149 participants) did not present any stage of the sarcopenic pathology, compared to 26.2% (53 subjects) who did have one of the stages related to this pathology (Figure 2). A total of 15 (7.4%) study participants with diagnosed sarcopenia were detected. The prevalence of sarcopenia in males was 29%, with eleven subjects having presented some of the stages related to sarcopenia according to the last diagnostic criteria of EWGSOP [3], and of these 3 cases (7.9%) were classified as diagnosed sarcopenia. Furthermore, 27 men (71%) did not meet the sarcopenic criteria. On the other hand, 25.6% of the women showed some stage of sarcopenia, compared to 74.4% without criteria. Of the first, 7.4% (12 women) were classified as diagnosed sarcopenia. 

The prevalence of diagnosed sarcopenia increased with age in women (*p* = 0.011); in contrast, among men, the differences between age groups in terms of the prevalence of the pathology were not significant (*p* = 0.196). For the whole sample, when the stratification by sex was not performed, significant differences were found in the prevalence of sarcopenia by age; thus, the group between 65 and 75 years had a prevalence of 3.1%, 14.9% being between 75 and 85 years (*p* = 0.004). This situation shows a clear relationship between age and sarcopenia in this study. No significant differences were found in the prevalence of total disease between the sexes (*p* = 1.000). The detailed distribution of the sample by gender, sarcopenic situation and age ranges, according to the criteria established by the 2019 EWGSOP [3], can be consulted in Table 1.

The different tests used to evaluate the presence of sarcopenic pathology yielded different results according to age, gender of the participants and the stage of the disease; all of them can be consulted in detail in Table 2. The differences in the results obtained for the different diagnostic tests of sarcopenia, between the group that presented the disease and the one that did not, were significant in the group of men except in the test of speed of march (*p* = 0.060) and the score obtained in the SARC-F questionnaire (*p* = 0.692). Among women, there were significant differences in all tests except the SARC-F questionnaire (*p* = 0.080). All the results of the diagnostic tests for sarcopenia obtained as a result of this study were lower in the group presenting the disease, with the exception of the score obtained in the SARC-F questionnaire, where the scores only showed a result ≥4 (indicative of disease) in 40% of the total cases of sarcopenia diagnosed in our study, and no significant differences were found between scores obtained in the sarcopenia probable and sarcopenia diagnosed groups (*p* = 0.15). 

Mean score and interpretation of SARC-F test according to sarcopenic status can be found in Table 3. 

## 4. Discussion

Our study provides information on the prevalence of sarcopenia in noninstitutionalized and autonomous older adults. The total prevalence of sarcopenia was found to be lower than those observed in other publications developed in Spain [39,40] in similar populations, among which there was a presence of the disease ranging from 10% to 11.8% in men, and between 22.9% and 33% in women. These publications followed the diagnostic criteria established by the EWGSOP in 2010 [15], and our results were compared with these data since no other known references were found in Spain that follow the criteria established by the EWGSOP in 2019 [3]. In other countries where the diagnostic criteria offered by the EWGSOP were followed in 2010, this trend was also observed; in the study by Moreira et al. [20] in Brazil, a prevalence of total sarcopenia was observed at 18%, and in the review carried out by Mayhew et al. [19], prevalence of the disease was observed at 36.7 and 62.2% in men and women, respectively. On the other hand, the results obtained in the work carried out by Kim et al. [41] in South Korea, under the criteria of the EWGSOP of 2019 [3], were similar to the results of our study. It is important to note that the publication developed by Kim et al. [41] was the only one found by the authors in which the diagnostic criteria of the EWGSOP 2019 [3] were used in noninstitutional elderly people. According to Cuesta et al. [39], we observed a higher prevalence of sarcopenia among women versus men, although our groups were not equitable, and Cuesta et al. enrolled more men in their sample. The significance between sexes could not be compared in other studies carried out with the criteria of EWGSOP 2019 [3], since this variable was not found to be represented.

Prevalence of this disease is higher in the institutionalized elderly [42,43] than our study. It was not possible to develop a comparison with the prevalence of sarcopenia in elderly patients institutionalized under the criteria of the EWGSOP 2019 [3], since no study was found in this population. According to Cuesta et al. [39], no significant relationship was found between the results obtained referring to ASMM and the grip strength regardless of gender (*p* = 0.285). Note that 17.3% of the sample showed low grip strength, compared to 26.7% with a low ASMM. The deficit of force in the upper train was slightly higher than that of the lower train (17.3% vs. 16.3%), we think that this situation may be due to the greater number of women who participated in the study (81.2%), as men seemed to retain more strength in the upper limbs. This is supported by numerous studies that analyze the grip strength in older people, although its purpose is not the diagnosis of sarcopenia [44,45].

Despite the fact that the SPPB test yielded worse results in the group with total sarcopenia compared to the group without the disease, regardless of sex, some remarkable findings were observed when comparing the results obtained between the different stages of sarcopenic pathology. Thus, in the case of women, worse results were found in the group with probable sarcopenia versus confirmed sarcopenia, regardless of age. This situation could be due to the fact that the strength deficit could play a more important role in the reality of sarcopenic pathology than the amount of muscle mass; therefore, it is possible that there were subjects with poorer physical condition but with an amount of muscle mass higher than the cutoff point that prevented them from progressing in the sarcopenic stages, acting as a bottleneck.

This situation leads us to the hypothesis that some subjects could present low strength and physical performance (worse SPPB) but, in turn, an amount of muscle mass (ASMM) still within the EWGSOP criteria, preventing categorization in more advanced stages of the pathology. These subjects could be in a situation of probable sarcopenia and not of confirmed or severe sarcopenia.

For the SARC-F questionnaire, none of the groups with a diagnosis of sarcopenia or probable sarcopenia obtained an average score ≥4, which leads us to question the effectiveness of the SARC-F questionnaire in the screening of sarcopenia; reflection that is also shared in the study carried out by Dodds et al. [46], which suggests a decrease in the value assigned to the cutoff point for the analysis of cases, with the aim of increasing the early detection of subjects with such pathology. Furthermore, in our study, the SARC-F test detected a higher percentage of false negatives in the diagnosed sarcopenia group (53.3%) compared to the probable sarcopenia group (34.2%), unlike what was expected in the consensus presented by the EWGSOP [3].

The variation in diagnostic criteria made by the EGWSOP in 2019 [3] may lead to a reduction in the diagnosis of sarcopenia; these, in turn, set a new standard for the calculation of the prevalence of the pathology. Our work presents methodological strengths of relevance: the diagnostic criteria of the EWGSOP published in 2019 have been fully respected [3]; they have also been presented, in a stratified manner, as data in stable age groups and with figures for all diagnostic tests involved in the diagnosis of sarcopenia.

The limitations of our study are that nonrandom sampling and the fact that it is a nonmulticentric study mean that the results cannot be representative or extrapolated to other populations. We consider it necessary to develop new publications that rigorously follow the algorithm proposed by the EWGSOP, with the aim of making comparisons between them and showing reliable results that are representative for the scientific community.

## 5. Conclusions

In conclusion, less than one in ten participants (7.4%) in the study had sarcopenia. The probable sarcopenia or risk of sarcopenia was present in 18.8% of the sample. The presence of sarcopenic pathology is greater as one advances in age, regardless of sex. The SARC-F questionnaire detected only 40% of the cases diagnosed with sarcopenia in this study. There is a need to develop new publications using the algorithm proposed by EWGSOP in 2019 and to allow comparisons to be made with the findings of this study.

## Figures and Tables

**Table 1:** Prevalence of sarcopenia in community-dwelling older adults by EWGSOP2 diagnosis criteria.

	**65–75 Years**	**75–85 Years**	**Total**
Men (n)	27	11	38
Without sarcopenia ^1^	21 (77.8%)	6 (54.5%)	27 (71%)
Total sarcopenia ^2^	6 (28.6%)	5 (45.4%)	11 (28.9%)
Sarcopenia probable ^3^	5 (18.5%)	3 (27.3%)	8 (21.1%)
Sarcopenia diagnosed ^4^ (SC ^5^ + SS ^6^)	1 (3.7%)	2 (18.2%)	3 (7.9%)
Sarcopenia confirmed ^7^	0 (0%)	0 (0%)	0 (%)
Sarcopenia severe ^8^	1 (3.7%)	2 (18.2%)	3 (7.9%)
Women (n)	101	63	164
Without sarcopenia ^1^	80 (79.2%)	42 (66.7%)	122 (74.4%)
Total sarcopenia ^2^	21 (20.8%)	21 (33.3%)	42 (25.6%)
Sarcopenia probable ^3^	18 (17.8%)	12 (19.0%)	30 (18.3%)
Sarcopenia diagnosed ^4^ (SC ^5^ + SS ^6^)	3 (3%)	9 (14.3%)	12 (7.3%)
Sarcopenia confirmed ^7^	2 (2%)	4 (6.3%)	6 (3.7%)
Sarcopenia severe ^8^	1 (1%)	5 (7.9%)	6 (3.7%)
Total (n)	128	74	202
Without sarcopenia ^1^	101 (78.9%)	48 (64.9%)	149 (73.8%)
Total sarcopenia ^2^	27 (21.1%)	26 (35.1%)	53 (26.2%)
Sarcopenia probable ^3^	23 (18%)	15 (20.3%)	18 (18.8%)
Sarcopenia diagnosed ^4^ (SC ^5^ + SS ^6^)	4 (3.1%)	11 (14.9%)	15 (7.4%)
Sarcopenia confirmed ^7^	2 (1.6%)	4 (5.4%)	6 (3%)
Sarcopenia severe ^8^	2 (1.6%)	7 (9.5%)	9 (4.5%)
	**65–75 years**	**75–85 years**	**Total**
Men (n)	27	11	38
Without sarcopenia ^1^	21 (77.8%)	6 (54.5%)	27 (71%)
Total sarcopenia ^2^	6 (28.6%)	5 (45.4%)	11 (28.9%)
Sarcopenia probable ^3^	5 (18.5%)	3 (27.3%)	8 (21.1%)
Sarcopenia confirmed ^4^	0 (0%)	0 (0%)	0 (0%)
Sarcopenia severe ^5^	1 (3.7%)	2 (18.18%)	3 (7.9%)
Sarcopenia diagnosed ^6^ (SC ^7^ + SS ^8^)	1 (3.7%)	2 (18.2%)	3 (7.9%)
Women (n)	101	63	164
Without sarcopenia ^1^	80 (79.2%)	42 (66.7%)	122 (74.4%)
Total sarcopenia ^2^	21 (20.8%)	21 (33.3%)	42 (25.6%)
Sarcopenia probable ^3^	18 (17.8%)	12 (19.0%)	30 (18.3%)
Sarcopenia confirmed ^4^	2 (2%)	4 (6.3%)	6 (3.7%)
Sarcopenia severe ^5^	1 (1%)	5 (7.9%)	6 (3.7%)
Sarcopenia diagnosed ^6^ (SC ^7^ + SS ^8^)	3 (3%)	9 (14.3%)	12 (7.3%)
Total (n)	128	74	202
Without sarcopenia ^1^	101 (78.9%)	48 (64.9%)	149 (73.8%)
Total sarcopenia ^2^	27 (21.1%)	26 (35.1%)	53 (26.2%)
Sarcopenia probable ^3^	23 (18%)	15 (20.3%)	18 (18.8%)
Sarcopenia confirmed ^4^	2 (1.6%)	4 (5.4%)	6 (3%)
Sarcopenia severe ^5^	2 (1.6%)	7 (9.5%)	9 (4.5%)
Sarcopenia diagnosed ^6^ (SC ^7^ + SS ^8^)	4 (3.1%)	11 (14.9%)	15 (7.4%)

^1^ muscle mass preserved with any degree of muscle dysfunction. ^2^ reduced muscle strength with preserved muscle quantity or quality and preserved physical performance. ^3^ reduced muscle strength with low muscle quantity or quality and preserved physical performance. ^4^ total cases of sarcopenia confirmed and sarcopenia severe. ^5^ sarcopenia confirmed. ^6^ sarcopenia severe. ^7^ reduced muscle strength with low muscle quantity or quality and low physical performance. ^8^ all cases with any degree of sarcopenic status.

**Table 2:** Results of the different tests to assess sarcopenia in groups established by EWGSOP2 ^1^.

	GS ^2^ (kg)	STS ^3^ (s)	ASMM Total ^4^ (kg)	Gait Speed ^5^	SPPB ^6^ (Score)	SARC-F (Score)
Men						
65–75 years						
Without Sarcopenia	36.5 ± 7.5	10.2 ± 2.4	23.2 ± 3.1	1.1 ± 0.2	10.3 ± 1.5	1.2 ± 1.4
Sarcopenia Probable	32.8 ± 5	16.2 ± 4.6	25 ± 1.3	0.9 ± 0.2	9 ± 2	2.6 ± 1.3
Sarcopenia Confirmed	-	-	-	-	-	-
Sarcopenia severe	25 ± 0	21 ± 0	16.9 ± 0	0.8 ± 0	5 ± 0	4 ± 0
Total Sarcopenia Diagnosed	25 ± 0	21 ± 0	16.9 ± 0	0.8 ± 0	5 ± 0	4 ± 0
Total Sarcopenia	28.9 ± 5.5	18.6 ± 3.4	20.9 ± 5.7	0.8 ± 0.1	7 ± 2.8	3.3 ± 0.9
75–85 years						
Without Sarcopenia	32.7 ± 4.5	12.6 ± 2.7	23 ± 2.5	0.9 ± 0.1	9.3 ± 2.2	3.2 ± 2.1
Sarcopenia Probable	28.7 ± 6.1	18.9 ± 1.4	24.5 ± 2.4	0.9 ± 0.3	5.3 ± 1.2	5 ± 2
Sarcopenia Confirmed	-	-	-	-	-	-
Sarcopenia severe	28 ± 1.4	16.5 ± 0	19.6 ± 0.3	0.8 ± 0.1	6.5 ± 0.7	1 ± 1.4
Total Sarcopenia Diagnosed	28 ± 1.4	16.5 ± 0	19.6 ± 0.3	0.8 ± 0.1	6.5 ± 0.7	1 ± 1.4
Total Sarcopenia	28.3 ± 0.5	17.7 ± 1.7	22 ± 3.5	0.8 ± 0.1	5.9 ± 0.8	3 ± 2.8
Women						
65–75 years						
Without Sarcopenia	22 ± 3.8	10.3 ± 2.1	16.8 ± 2.4	1.1 ± 0.2	10.4 ± 1.4	1.7 ± 1,2
Sarcopenia Probable	16.5 ± 5.8	14.1 ± 4.3	17.9 ± 2	1 ± 0.3	8.7 ± 1.7	3.2 ± 1.5
Sarcopenia Confirmed	12.7 ± 3.2	11.6 ± 1.1	14.2 ± 2.2	1 ± 0.1	9.7 ± 2.1	1.3 ± 1.5
Sarcopenia severe	15 ± 0	19.3 ± 0	14.4 ± 0	0.7 ± 0	5 ± 0	4 ± 0
Total Sarcopenia Diagnosed	13.9 ± 1.6	15.5 ± 0.6	14.3 ± 1.1	0.9 ± 0.1	7.4 ± 1.1	2.7 ± 0.8
Total Sarcopenia	14.7 ± 1.9	15 ± 3.9	15.5 ± 2.1	0.9 ± 0.2	7.5 ± 2.1	3.5 ± 0.5
75–85 years						
Without Sarcopenia	20.2 ± 3.6	11 ± 2	15.9 ± 2.2	1 ± 0.2	10 ± 1.4	2.6 ± 1.3
Sarcopenia Probable	14.9 ± 3	17.2 ± 4.8	17.7 ± 2.4	0.7 ± 0.2	7.4 ± 1.7	4.1 ± 1
Sarcopenia Confirmed	14.8 ± 1.5	12.7 ± 1.7	13 ± 2.4	0.9 ± 0.1	10.3 ± 1.5	3.8 ± 0.5
Sarcopenia severe	12.6 ± 1.3	15.3 ± 4.6	13.9 ± 1.7	0.8 ± 0.2	7.8 ± 1.1	2.8 ± 2.3
Total Sarcopenia Diagnosed	13.7 ± 1.4	14 ± 3.2	13.5 ± 2.1	0.9 ± 0.2	9.1 ± 1.3	3.3 ± 1.4
Total Sarcopenia	14.1 ± 1.3	15.1 ± 2.3	14.9 ± 2.5	0.8 ± 0.1	8.5 ± 1.6	3.6 ± 0.7
Men						
WS ^7^ (all sample sizes)	34.6 ± 6	11.4 ± 2.6	23.1 ± 2.8	1 ± 0.2	9.8 ± 1.9	2.2 ± 2.8
TSD ^8^ (all sample sizes)	26.5 ± 0.7	18.8 ± 0	18.3 ± 0.15	0.8 ± 0.1	5.8 ± 0.4	2.5 ± 0.7
TS ^9^ (all sample sizes)	28.63 ±0.4	18.15 ± 0.6	21.5 ± 0.8	0.85 ± 0.0	6.45 ± 0.8	3.15 ± 0.2
Women						
WS ^7^ (all sample sizes)	21.1 ± 3.4	10.7 ± 2.1	16.4 ± 2.3	1.1 ± 0.2	10.2 ± 1.4	2.2 ± 1.3
TSD ^8^ (all sample sizes)	13.8 ± 1.5	14.8 ± 1.9	13.9 ± 1.6	0.9 ± 0.2	8.3 ± 1.2	3 ± 1.1
TS ^9^ (all sample sizes)	14.4 ± 0.4	15 ± 0	15.2 ± 0.4	0.9 ± 0.1	8 ± 0.7	3.5 ± 0.1

^1^ Values are presented as mean ± standard error. ^2^ Grip Strength. ^3^ Sit-To-Stand Test. ^4^ Appendicular Skeletal Muscle Mass. ^5^ Gait Speed. ^6^ Short Physical Performance Battery. ^7^ Without Sarcopenia. ^8^ Total Sarcopenia Diagnosed. ^9^ Total Sarcopenia.

**Table 3:** Mean score and interpretation of SARC-F test according to sarcopenic status ^1.^

	Total (n)	SARC-F Score	Positive SARC-F (≥4)	Negative SARC-F (<4)
Without sarcopenia ^2^	149	1.95 ± 1.42	23 (15.4%)	126 (84.6%)
Total sarcopenia ^3^	53	2.93 ± 1.46	32 (60.4%)	21 (39.6%)
Sarcopenia probable ^4^	38	2.5 ± 1.52	25 (65.8%)	13 (34.2%)
Sarcopenia diagnosed ^5^ (SC ^6^ + SS ^7^)	15	2.8 ± 1.78	7 (46.7%)	8 (53.3%)

^1^ Values are presented as mean ± standard deviation, n or n (%). ^2^ muscle mass preserved with any degree of muscle dysfunction. ^3^ reduced muscle strength with preserved muscle quantity or quality and preserved physical performance. ^4^ reduced muscle strength with low muscle quantity or quality and preserved physical performance. ^5^ total cases of sarcopenia confirmed and sarcopenia severe. ^6^ sarcopenia confirmed. ^7^ sarcopenia severe.
